# Myasthenia Crisis as First Presentation of MuSk Positive Myasthenia Gravis: A Case Report

**DOI:** 10.31729/jnma.8896

**Published:** 2025-03-31

**Authors:** Dipesh Chhetri, Amit Sharma Bhattarai, Pankaj Joshi, Kriti Thapa

**Affiliations:** 1 Chirayu National Hospital and Medical Institute, Basundhara, Kathmandu, Nepal; 2 Department of Anaesthesiology, Maharajgunj Medical Campus, Maharajgunj, Kathmandu, Nepal

**Keywords:** *case reports*, *MuSK*, *myasthenia crisis*, *myasthenia gravis*

## Abstract

We report a unique presentation of a 57-year-old female presented with severe respiratory acidosis, which was found to be a case of Musk-positive Myasthenia Gravis. Patient presented with depressed level of consciousness and respiratory failure, mandating urgent ventilatory support. She responded well with positive pressure ventilation. However, she persistently failed to maintain adequate ventilation after extubation and had to be reintubated. At presentation, the absence of classical symptoms typical of Myasthenia Gravis posed a diagnostic dilemma, initially obscuring the underlying etiology. However, since there was no other identifiable cause for the inability to maintain ventilation, antibody panels were sent which turned out positive for Muscle specific kinase. This case thus highlights the significance of considering atypical neuromuscular presentations, particularly when respiratory compromise is the predominant manifestation, highlighting the necessity for comprehensive neurological evaluation even in the absence of classical symptoms for timely diagnosis and management of Myasthenia Gravis.

## INTRODUCTION

Myasthenia crisis is a sudden exacerbation of a myasthenia gravis characterized by respiratory fatigue and leading to respiratory failure urgently requiring ventilatory support to prevent death. Myasthenia crisis occurs in 15-20% of patients with myasthenia gravis, most within the first 2 years of diagnosis.^1^ In about 20% of patients, myasthenia crisis is the first manifestation of yet undiagnosed myasthenia gravis.^1^ However, Respiratory failure was the initial presentation in only 2% of patients with MuSK-positive Myasthenia Gravis, highlighting its rarity as a presenting feature.^2^ This case underscores the clinical variability of MuSK-positive MG, presenting with a myasthenic crisis and respiratory failure as the initial manifestation. While ptosis and bulbar symptoms were later elicited, their subtle nature delayed diagnosis. Furthermore, the presence of comorbidities such as diabetes, hypertension, subclinical hypothyroidism, schizophrenia and concurrent lower respiratory tract infection likely contributed to diagnostic challenges, as these conditions can mimic or obscure MG symptoms and complicate clinical assessment.^3^

## CASE REPORT

A 57-year-old female presented to the emergency department in November 2023 with an hour-long loss of consciousness, altered sensorium, irritability, refusal to eat or drink, and loss of bowel and bladder control for a day. Her medical history included pulmonary embolism (6 months prior, treated with Rivaroxaban), schizophrenia, diabetes, and hypertension, all controlled under medications.

On examination, she had GCS of E1V1M1, accompanied by reduced chest movements and diminished breath sounds on auscultation. Arterial blood gas analysis showed significant respiratory acidosis with pH 7.1 and pCO2 129.9. The patient was immediately intubated and put on mechanical ventilation. A thorough blood analysis revealed Hb at 11.6, WBC at 10,700, Sodium at 132, Potassium at 4.5, decreased serum calcium at 7.5, Urea/Creatinine at 36/1.6, ESR at 19, and an elevated RBS of 395 mg/dl with 2+ sugar in the urine. Urine acetone was negative. She was started on IV fluids alongside Inj Noradrenaline infusion to manage her hypotension while withholding all her antihypertensives. Concurrently, IV antibiotics Piperacillin/Tazobactam and Levofloxacin were commenced as a part to manage her lower respiratory tract infection. Over next 2 hours, she started showing signs of neurological improvement and subsequent ABG post mechanical ventilation demonstrated significant improvement in acid-base status with pH corrected to 7.34 and pCO2 reduced to 60mmHg.

Noradrenaline was tapered and stopped by the evening on the same day. She was extubated the next morning and was started on non-invasive ventilation (NIV). She was initially stable on NIV, however, her condition deteriorated the next day with oxygen desaturation, bradycardia, and altered consciousness, leading to cardiac arrest. CPR was initiated along with airway control, with return of spontaneous circulation within a minute.

When we repeatedly inquired about her symptoms to the family, they provided us with a subtle history of ptosis that becomes prominent during the evening, dysarthria and difficulties with chewing and swallowing, all of which represented the bulbar symptoms. An antibody panel revealed negative Acetylcholine receptor binding (AChR) Antibody, but was positive for ANA and Muscle Specific Kinase (MuSK) Antibody.

She was then started on steroids Inj Hydrocortisone 50mg QID. The next day, she started showing improvements in neurological and respiratory parameters. She was extubated the next day following significant improvement in blood gas. Non-Invasive Mechanical Ventilation was initiated following extubation. She was gradually weaned off NIV to facemask and then to nasal cannula.

She was then started on Tab pyridostigmine 30mg BD and Tab Prednisolone in the tapering dose while stopping Inj hydrocortisone. Additionally, paraneoplastic panel was also sent, which yielded negative results.

She began pulmonary rehabilitation with incentive spirometry, and oxygen therapy was gradually tapered. She maintained normal CO2 levels, and after 19 days in the ICU and general ward, was discharged. Her plan included repeat blood gas tests, continued pyridostigmine, tapering prednisolone, and follow-up pulmonary function test, with a plan to start Azathioprine.

## DISCUSSION

Myasthenia gravis is a rare auto-immune disorder characterized by the formation of antibodies against the nicotinic acetylcholine receptors at the neuromuscular junction or other postsynaptic muscle fibre components like muscle specific tyrosine kinase (MuSK). MuSk Antibodies were first time identified in a myasthenia gravis patient in 2001.^4^ Although rare, myasthenia gravis is the most common disorder of the neuromuscular junction, with an annual incidence of 0.25-2 patients per 100,000,5 and it is estimated to affect more than 700,000 patients worldwide.^6^

It is recognized that the type of antibodies present in the disease determines it's clinical behavior.^7^ Myasthenia patients with antibodies against acetylcholine receptors (AChR) have more of a variable presentation which includes weakness in the limbs more than the bulbar muscles involvement, marked ptosis, and associated thymoma in 80% of the cases.^7^ Whereas, myasthenia patients who have antibodies against MuSk, 80% of them show bulbar impairment,^4^ including dysphagia, dysphonia, chewing problems, slurred speech and tongue weakness/atrophy. Our patient, although didn't have muscle atrophy, but had other subtle bulbar symptoms. MuSk positive myasthenia are less commonly associated with thymoma or thymic hyperplasia and may show poor response to conventional therapy including thymectomy and pyridostigmine. However, more than 50% patients show symptomatic stability and remission when treated with a combination of AChE-I like pyridostigmine and a steroid.^8^ In addition, they are more prone to myasthenia crisis.^9^

Myasthenia crisis is a life-threatening complication of Myasthenia gravis. Early intubation and mechanical ventilation forms the mainstay in the management of myasthenia crisis.^1^ Post extubation NIV can be used to prevent re-intubation in Myasthenia crisis.^10^ Besides early mechanical ventilation, specific immunotherapy consisting of Plasmapheresis or Human IVIg is considered as a part of standard of care in patients with myasthenia crisis,^10^ and results in improvement in almost 70% of myasthenic patients with severe weakness.^1^ In our patient, plasmapheresis or IVIg was not considered to begin with due to the initial lack of suspicion of MG, with the acute presentation dominated by metabolic acidosis, sepsis, and comorbid conditions. Following confirmation of MuSK antibody positivity, corticosteroid therapy was initiated, resulting in rapid clinical improvement and therefore negating the need for plasmapheresis or IVIg.

Although myasthenia crisis can occur spontaneously in 1/3rd of the patients, the most common precipitant is infection, most commonly the bacterial pneumonia.^5^ Besides, other triggers being reduction of medication dose, pregnancy, perimenstrual period and certain medications like antibiotics (aminoglycosides, macrolides, fluoroquinolones, polymyxin), antiepileptics (phenytoin, gabapentin) and steroids.^5,11^ Steroid might sound paradoxical as steroids are also used in the management of Myasthenia crisis, but the initial treatment with steroids can lead to worsening symptoms in almost half of the patients and can lead to myasthenia crisis in 9-18% of patients.^12^ However, our case highlights a rapid and favourable response to early steroid initiation. This suggests that in selected cases, particularly under close monitoring and with the appropriate clinical context, steroids can be an effective initial treatment, potentially circumventing the need for more invasive therapies like IVIG or plasmapheresis.

The median duration of hospital stay in patients with myasthenia crisis is about 17-35 days.^1^ The mortality rate in myasthenia crisis patients presenting to hospital lies around 5%,^1^ however, it stands around 9.5% for MuSK positive myasthenia crisis.^8^ The cause of death being primarily sepsis, cardiac arrest, multiple organ failure and ARDS.^1^
